# Exploring the tradeoff between data privacy and utility with a clinical data analysis use case

**DOI:** 10.1186/s12911-024-02545-9

**Published:** 2024-05-30

**Authors:** Eunyoung Im, Hyeoneui Kim, Hyungbok Lee, Xiaoqian Jiang, Ju Han Kim

**Affiliations:** 1 College of Nursing, Seoul National University, Seoul, South Korea; 2 Center for World-leading Human-care Nurse Leaders for the Future by Brain Korea 21 (BK 21) four project, College of Nursing, Seoul National University, Seoul, South Korea; 3 The Research Institute of Nursing Science, Seoul National University, Seoul, South Korea; 4 School of Biomedical Informatics, UTHealth, Houston, TX USA; 5 Seoul National University Hospital, Seoul, South Korea; 6 College of Medicine, Seoul National University, Seoul, South Korea

**Keywords:** Data privacy, Data utility, Data de-identification, Clinical data analysis, ARX tool

## Abstract

**Background:**

Securing adequate data privacy is critical for the productive utilization of data. De-identification, involving masking or replacing specific values in a dataset, could damage the dataset’s utility. However, finding a reasonable balance between data privacy and utility is not straightforward. Nonetheless, few studies investigated how data de-identification efforts affect data analysis results. This study aimed to demonstrate the effect of different de-identification methods on a dataset’s utility with a clinical analytic use case and assess the feasibility of finding a workable tradeoff between data privacy and utility.

**Methods:**

Predictive modeling of emergency department length of stay was used as a data analysis use case. A logistic regression model was developed with 1155 patient cases extracted from a clinical data warehouse of an academic medical center located in Seoul, South Korea. Nineteen de-identified datasets were generated based on various de-identification configurations using ARX, an open-source software for anonymizing sensitive personal data. The variable distributions and prediction results were compared between the de-identified datasets and the original dataset. We examined the association between data privacy and utility to determine whether it is feasible to identify a viable tradeoff between the two.

**Results:**

All 19 de-identification scenarios significantly decreased re-identification risk. Nevertheless, the de-identification processes resulted in record suppression and complete masking of variables used as predictors, thereby compromising dataset utility. A significant correlation was observed only between the re-identification reduction rates and the ARX utility scores.

**Conclusions:**

As the importance of health data analysis increases, so does the need for effective privacy protection methods. While existing guidelines provide a basis for de-identifying datasets, achieving a balance between high privacy and utility is a complex task that requires understanding the data’s intended use and involving input from data users. This approach could help find a suitable compromise between data privacy and utility.

**Supplementary Information:**

The online version contains supplementary material available at 10.1186/s12911-024-02545-9.

## Background

Clinical data gathered through Electronic Health Records (EHR) is an invaluable asset for producing meaningful insights into patient care and healthcare service management. However, as this data includes sensitive personal information, there is a heightened risk of financial or social damage to individuals if their health data is improperly disclosed [1, 2]. To address these concerns, many countries have implemented stringent regulations to safeguard patient privacy while still enabling the efficient use of data for health advancements [3]. In the United States, for example, the Health Insurance Portability and Accountability Act (HIPAA) sets forth provisions for data protection and usage [4]. Similarly, the General Data Protection Regulation (GDPR) offers a comprehensive data privacy framework within the European Union [5]. Additionally, South Korea’s Personal Information Protection Act delineates the guidelines for secure and permissible data handling [6].

The growing imperative for data privacy has spurred significant progress in privacy-preserving technologies. Differential Privacy (DP) safeguards data by integrating controlled random noise, thus ensuring individual data points remain confidential while aggregate analysis remains accurate [7]. In the biomedical field, DP is extensively employed in data query systems; the noise integrated into query responses helps protect sensitive inquiries pertaining to uncommon cases [8, 9]. Current research in DP focuses on solving complex problems such as determining optimal privacy budgets and noise levels to balance confidentiality with data utility [8, 10, 11].

Homomorphic Encryption (HE) represents a breakthrough in cryptography for preserving privacy, enabling computations on encrypted data without altering the original values [12]. Recent research has validated the practicality of performing data analysis using HE [13–15]. Nonetheless, HE has not become mainstream in healthcare applications, primarily due to its substantial computational demands, intricate implementation, and the limited range of analytics that can be performed on data in its encrypted form [12, 16].

Blockchain technology, recognized for its immutable, decentralized, and transparent nature [17], is gaining attention as an innovative approach for data privacy [18–20]. Despite this interest, the real-world application of blockchain is contingent upon enhancements in its capacity to process substantial data volumes, simplification of its implementation, and resolution of related regulatory challenges [21–24].

When preparing datasets with personal health information for secondary analysis, the prevailing practice is to mitigate the risk of re-identification of the subjects in the dataset by employing stringent de-identification procedures [25, 26]. This involves the removal of direct identifiers that can uniquely pinpoint individual subjects within the dataset and altering quasi-identifiers, which alone do not identify subjects but could do so when merged with other data sources. Furthermore, the process considers sensitive information that, despite not directly identifying subjects, could have detrimental effects if disclosed, ensuring such data is also considered during the de-identification process.

The leading method for data de-identification employs strategies like K-anonymity, L-diversity, and T-closeness to modify data. K-anonymity safeguards against linkage attacks by ensuring that there are at least K identical records for any set of quasi-identifiers within a dataset, making it impossible to distinguish one individual from K-1 others [27]. In line with this, South Korea’s data publishing guidelines recommend adhering to a minimum of ‘K = 3’ for K-anonymity [28, 29]. Additionally, L-diversity mandates a sensitive variable must have at least L distinct values, thereby offering protection against homogeneity attacks [30]. T-closeness, on the other hand, ensures that the distribution of a sensitive variable within any subset of the dataset closely approximates the distribution of that variable of the entire dataset, adhering to a specified threshold [31]. T-closeness prevents the likelihood that knowledge of the variable’s distribution could be exploited to reveal an individual’s identity [31]. The process of de-identification, which often involves masking or altering certain data values, can result in information loss and potentially reduce the utility of the dataset [32].

Determining the optimal threshold between data privacy and utility remains a complex challenge. Several studies have investigated how various de-identification strategies, specifically K-anonymity, L-diversity, and T-closeness, influence data utility. This is typically assessed by comparing the analytical results of de-identified datasets with those derived from the original dataset. Some researchers advocate that the privacy enhancements are overshadowed by a substantial reduction in data utility [33, 34], while others argue that such utility loss might not be as severe as some studies imply [35]. However, these studies evaluated each de-identification technique in isolation, often resorting to simplified models that fail to fully capture the complexities of real-world data use, and led to mixed conclusions [34, 35].

Moreover, the insights offered by such research into the tangible effects of data de-identification on actual data analysis tasks are somewhat restricted. This is because the analyses were either performed using overly simplistic examples [28, 34] or on public datasets that have already undergone some form of de-identification [35, 36], or focusing on theoretical aspects [37]. Therefore, there is a need for more intricate research that closely mirrors the complexities of real-life data analytics tasks and considers the multifaceted nature of data utility and privacy in actual applications.

This study explores the effects of different de-identification strategies on clinical datasets prepared for secondary analysis, with a focus on their implications for practical data analysis tasks. The aims of this study are twofold: firstly, to assess the effects of de-identification on both the dataset’s integrity and the outcomes of data analyses; and secondly, to ascertain if discernible trends emerge from the application of various de-identification techniques that could guide the establishment of a feasible balance between data privacy and data utility.

## Methods

### Data analysis use case

This study explores the impact of various de-identification techniques on datasets and their subsequent analysis results using a data analytic use case. The analytic use case involved predicting the Length of Stay (LOS) of high-acuity patients transferred to the emergency department (ED) of an academic medical center located in Seoul, South Korea. LOS in the ED serves as a crucial quality metric for ED services [38–40]. In Korea, an ED LOS under six hours is considered optimal [41]. Nonetheless, the overcrowding issues prevalent in tertiary hospital EDs elevate the risk of prolonged ED stays for patients transferred from other facilities for specialized care [42, 43]. Understanding the factors affecting the ED LOS of transferred high-acuity patients is essential to providing timely care. The authors, HK and HL, previously developed a model to predict ED LOS using logistic regression, Random Forest, and Naïve Bayes techniques [44]. Building on insights from this earlier research, the current use case was crafted to develop a logistic regression model to predict ED LOS based on variables including the patient’s sex, age, medical conditions, the type and location of the transferring hospital, and the treatment outcomes.

### Dataset

The prediction model for ED LOS was developed using data from 1,155 patients who were transferred to the study site’s ED between January 2019 and December 2019. Patient demographics, clinical details, and transfer-related information were extracted from the study site’s Clinical Data Warehouse (CDW). The variables collected for this study are listed in Table 1.

**Table 1:** The variables extracted from the clinical data warehouse

Variables	Description
Sex	Sex of the patients
Age	Age of the patients in years
Acuity level	Patient’s acuity level classified based on Korean Triage and Acuity Scale (KTAS) level
Number of consults	Number of consults requested from the emergency department
Inter-hospital communication	Prior communication between medical staff at the time of transfer
Sending hospital	Name and address of the healthcare facility that the patient was transferred from
Primary diagnosis	Patients’ main diagnosis coded with International Classification of Diseases (ICD 10)
Treatment outcome	Follow-up measures according to patients’ progress after admission to the emergency department (e.g., Discharge, Admission, Transfer)
Length of stay	Duration that the patient stayed at ED, measured in hours

### De-identification of the datasets

#### Developing de-identification scenarios

Identifiers such as patient names and medical record numbers were removed. Quasi-identifiers play a critical role in de-identification as they form the foundation for assessing the adequacy of de-identification efforts and undergo most data transformations. To select the variables to test as quasi-identifiers, we first examined the extent to which each variable could uniquely link to individual subjects within the dataset, potentially identifying them. Table 2 displays the percentage of subjects in the dataset uniquely linked to either a single variable or a combination of variables. For instance, the *sending hospital* and *primary diagnosis* were uniquely linked to 27.71% and 17.75% of the subjects, respectively, and their combination linked up to 94% of the subjects. Consequently, information regarding the *sending hospital* and the *primary diagnosis*, coded using the International Classification of Disease (ICD) [45], were utilized as quasi-identifiers, along with *sex* and *age*, which are commonly considered quasi-identifiers in various de-identification efforts [4, 46]. Treatment outcomes were identified as sensitive information. We developed 19 de-identification scenarios by varying the quasi-identifiers and sensitive information, and applying diverse configurations of privacy-preserving techniques such as K-anonymity, L-diversity, and T-closeness to each scenario.

**Table 2:** The fraction of the records potentially identifiable by single or combinations of variables

Single or combinations of variables	% Records potentially identifiable
Sex	0%
Age	0.52%
Primary diagnosis	17.75%
Sex + Primary diagnosis	25.02%
Sending hospital	27.71%
Sex + Sending hospital	38.10%
Sex + Age + Primary diagnosis	83.72%
Age + Sending hospital	88.40%
Primary diagnosis + Sending hospital	93.59%
Age + Sending hospital + Primary diagnosis	98.70%
Sex + Age + Sending hospital + Primary diagnosis	98.70%

#### Data transformation for de-identification

De-identification was performed using ARX, a publicly accessible and well-validated data anonymization tool that supports various de-identification methods [47–49]. We employed generalization and micro-aggregation techniques to modify the quasi-identifiers, both aimed at reducing the risk of re-identification by transforming original data into more general values. Generalization involves building a hierarchy for the given values by specifying minimum and maximum generalization levels. Generalization involves creating a hierarchy of values by specifying minimum and maximum levels, which can be adjusted based on criteria such as the number of digits masked in zip codes, size of intervals for *age*, condensation of 5-point Likert scores to 3-point scales, and generalization of full dates to broader time units such as week, month, or year [50]. Micro-aggregation, on the other hand, assigns representative values for alphanumeric data, such as using the mode for *sex* and the mean for *age* [50].

In our de-identification process, quasi-identifiers such as the *sending hospital* and *primary diagnosis* were transformed using generalization, while *sex* was modified through micro-aggregation. *Age* was subjected to both generalization and micro-aggregation. The generalization hierarchy for *age* included three levels with intervals of 5, 10, and 30 years respectively. For micro-aggregation, mean *age* values were used. The *primary diagnosis* was generalized into two levels based on higher-level ICD codes. For instance, a *primary diagnosis* with the ICD code I20.0, representing *unstable angina*, was generalized to I20 (i.e., *angina pectoris*) at level 1, and further to I20-I25 (i.e., *ischemic heart diseases*) at level 2. Generalization of the *sending hospital* also included two levels, where a specific facility such as “Hanmaeum Clinic in Jongno-gu, Seoul city” was generalized to the county level as “facility in Jongno-gu” at level 1 and then to the city level as “facility in Seoul” at level 2. For *sex*, micro-aggregation was employed, setting the mode as the representative value.

K-anonymity, L-diversity, and T-closeness were employed concurrently with specific parameters set for each: K and L were both set at 3, and T was set at 0.5. K-anonymity was specifically applied to quasi-identifiers to ensure that each individual is indistinguishable from at least two others. L-diversity and T-closeness, on the other hand, were applied to the variable designated as sensitive, ensuring that sensitive information is both sufficiently diverse and closely aligned with the overall distribution of the dataset. Table 3 details these 19 de-identification scenarios.

**Table 3:** Data de-identification scenarios

De-identification scenario	Sex	Age	Primary diagnosis	Sending hospital	Treatment outcome
1	Micro-aggregation (mode)	Micro-aggregation (mean)	Generalization	Generalization	L-diversity
2	Micro-aggregation (mode)	Generalization	Generalization	Generalization	L-diversity
3	Micro-aggregation (mode)	Micro-aggregation (mean)	Generalization	Generalization	L-diversity, T-closeness
4	Micro-aggregation (mode)	Generalization	Generalization	Generalization	L-diversity, T-closeness
5	Micro-aggregation (mode)	Micro-aggregation (mean)	Generalization	L-diversity	L-diversity
6	Micro-aggregation (mode)	Generalization	Generalization	L-diversity	L-diversity
7	Micro-aggregation (mode)	Micro-aggregation (mean)	Generalization	L-diversity, T-closeness	L-diversity, T-closeness
8	Micro-aggregation (mode)	Generalization	Generalization	L-diversity, T-closeness	L-diversity, T-closeness
9	Micro-aggregation (mode)	Micro-aggregation (mean)	L-diversity	Generalization	L-diversity
10	Micro-aggregation (mode)	Generalization	L-diversity	Generalization	L-diversity
11	Micro-aggregation (mode)	Micro-aggregation (mean)	L-diversity, T-closeness	Generalization	L-diversity, T-closeness
12	Micro-aggregation (mode)	Generalization	L-diversity, T-closeness	Generalization	L-diversity, T-closeness
13	Micro-aggregation (mode)	Generalization	L-diversity	L-diversity	L-diversity
14	Micro-aggregation (mode)	Generalization	L-diversity, T-closeness	L-diversity, T-closeness	L-diversity, T-closeness
15	Micro-aggregation (mode)	Generalization	-	-	L-diversity
16	Micro-aggregation (mode)	Generalization	-	-	L-diversity, T-closeness
17	Micro-aggregation (mode)	-	Generalization	Generalization	L-diversity, T-closeness
18	-	Generalization	Generalization	Generalization	L-diversity, T-closeness
19	-	-	Generalization	Generalization	L-diversity, T-closeness

Data transformation was carried out in ARX according to the de-identification scenarios outlined in Table 3. ARX provides options to adjust additional transformation parameters: the *suppression limit*, which sets the maximum proportion of records that can be omitted from the original dataset; *approximation*, which prioritizes solutions with shorter execution times; and *precomputation*, which determines the threshold for the fraction of unique data values in the dataset [50]. For this study, we utilized the default settings in ARX, where the *suppression limit* was set to 100%, and both *approximation* and *precomputation* features were disabled.

**Fig. 1:**
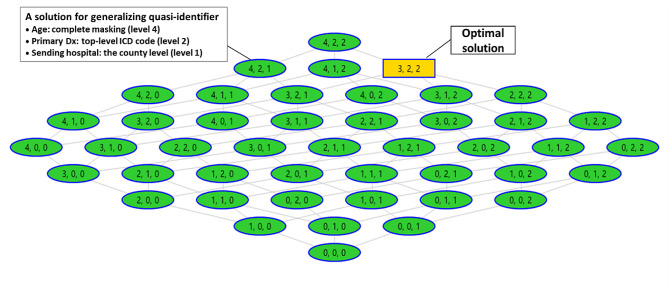
The data transformation solutions suggested by ARX

During execution, ARX evaluated various combinations of generalization and micro-aggregation levels to meet the requirements for K-anonymity, L-diversity, and T-closeness, ultimately recommending an optimal solution based on the balance between minimizing re-identification risk and preserving data utility. Figure 1 displays a screenshot of the data transformation solutions for the scenario where *age*, *primary diagnosis*, and *sending hospital* were designated as quasi-identifiers. Ultimately, we produced 19 versions of de-identified datasets, each based on the transformation solution that ARX identified as optimal.

#### Examination of the de-identified datasets

We reviewed the reduction in re-identification risk and the data utility scores that ARX estimated for the 19 de-identified datasets. To assess the similarity between each de-identified dataset and the original dataset, we employed Earth Mover’s Distance (EMD) [51]. Additionally, we calculated the dataset retention ratio. This metric is derived by dividing the number of data points in the transformed dataset by the number of data points in the original dataset. EMD and dataset retention ratio quantitatively evaluate the dissimilarity between the original dataset and the de-identified datasets, offering insights into how much the data has been altered through de-identification.

### Testing the effects of de-identification on ED LOS prediction

#### Variable creation for predictive modeling

To construct a logistic regression model for predicting ED LOS, we defined outcome and predictor variables. ED LOS, the outcome variable, was dichotomized into two categories: 6 h or less, and more than 6 h. We identified 13 predictors, including patient sex, age, medical conditions, treatment outcome, and the sending hospital type. *Age*, *sending hospital location*, and *treatment outcome* were dichotomized. Five dummy variables were created from *primary diagnosis* to represent *high priority disease*, *neoplastic disease*, *circulatory disease*, *respiratory disease*, and *injury-related visits*. The *sending hospital type* was derived from the *sending hospital information*. These variables, detailed in Table 4, were consistently defined across all 19 de-identified datasets as well as the original dataset to facilitate comparative analyses.

**Table 4:** The definitions of the variables used in the logistic regression analyses

Variable names	Descriptions (encoding)
Sex	Sex of the patients (0 = Male, 1 = Female)
Age	Age of the patients in years (0 = < 60yrs, 1 = ≥ 60yrs)
Acuity level	Severity classification according to Korean Triage and Acuity Scale (KTAS) level (0 = Level 1, 1 = Others)
Number of consults	The number of consults (0 = < 3, 1 = ≥ 3)
Inter-hospital communication	Prior communication between medical staff at the time of transfer (0 = No, 1 = Yes)
Sending hospital location	Location of the sending hospital (0 = Inside seoul, 1 = Outside seoul)
High priority disease	Classification of high priority diseases included in the Korean High Priority Diseases Classification Standards (0 = No, 1 = Yes)
Neoplastic disease	Presence of neoplasm diseases (0 = No, 1 = Yes)
Circulatory disease	Presence of circulatory diseases (0 = No, 1 = Yes)
Respiratory disease	Presence of respiratory diseases (0 = No, 1 = Yes)
Sending hospital type	Type of the sending hospital according to patient’s length of stay (0 = Short-term care facility, 1 = Long-term care facility)
Injury-related visits	Reason/types for visiting the emergency room due to illness or injury (0 = Disease, 1 = Injury)
Treatment outcome	Whether additional plans were established for patient treatment results (0 = Discharge/ Against medical advice (AMA) discharge, 1 = Admission/Procedure/Operation/Transfer to other hospitals)
ED LOS	The length of stay at Emergency Department (0: LOS <= 6 h, 1: LOS > 6 h)

#### Data analysis

After defining the outcome and predictor variables for logistic regression, we examined their distributions across the 19 de-identified datasets and the original dataset. To assess the differences in variable distributions, we utilized the proportion test [52]. Subsequently, logistic regression analysis was conducted using both the de-identified and original dataset. The predictive performance of these models was evaluated using the Area Under the Curve (AUC) of the Receiver Operating Characteristic (ROC) curve. We compared the AUC scores (AUROC) of the logistic regression models derived from the 19 de-identified datasets to that from the original dataset, employing the DeLong test [53]. Additionally, we analyzed the differences in the odds ratios of the predictors and their statistical significance to assess any impact the de-identification process might have had on the predictive capability of the models. All analyses were performed using R (version 4.0.4) [54].

## Results

### Data transformation configurations applied for the de-identification of the datasets

Table 5 displays the optimal configurations for data transformation used in the 19 de-identified datasets. Variables subjected to generalization or micro-aggregation were designated as quasi-identifiers. Sensitive information is identified as ‘SI’ within the table. It is important to note that empty cells signify that the corresponding variable was treated as non-sensitive information in the specific dataset.

**Table 5:** The data transformation configurations applied to the de-identified datasets

De-identified dataset numbers	Sex	Age	Primary diagnosis	Sending hospital	Treatment outcome
1	Micro -mode	Micro -mean	Gen -level 2	Gen -level 2	SI
2	Micro -mode	Gen -level 3	Gen -level 2	Gen -level 2	SI
3	Micro -mode	Micro -mean	Gen -level 2	Gen -level 2	SI
4	Micro -mode	Gen -level 3	Gen -level 2	Gen -level 2	SI
5	Micro -mode	Micro -mean	Gen -level 2	SI	SI
6	Micro -mode	Gen -level 3	Gen -level 2	SI	SI
7	Micro -mode	Micro -all masking	Gen -all masking	SI	SI
8	Micro -mode	Gen -all making	Gen -all masking	SI	SI
9	Micro -mode	Micro -mean	SI	Gen -level 1	SI
10	Micro -mode	Gen -level 3	SI	Gen -level 1	SI
11	Micro -mode	Micro -mean	SI	Gen -level 2	SI
12	Micro -mode	Gen -level 3	SI	Gen -level 2	SI
13	Micro -mode	Gen -level 1	SI	SI	SI
14	Micro -mode	Gen -level 3	SI	SI	SI
15	Micro -mode	Gen -level 1			SI
16	Micro -mode	Gen -level 1			SI
17	Micro -mode		Gen -level 2	Gen -level 2	SI
18		Gen -level 3	Gen -level 2	Gen -level 2	SI
19			Gen -level 2	Gen -level 2	SI

*Note.* Micro: micro-aggregation, Gen: generalization, SI: sensitive information

### The de-identified datasets

Table 6 displays the re-identification reduction rates, ARX utility scores, EMD scores, and dataset retention ratios for the 19 transformed datasets. Additionally, the table presents the number of records retained post-transformation and the number of predictor variables generated. The ARX utility score reflects the extent of information loss, with a higher score indicating lower utility. It is important to note that the baseline re-identification risk varied among the datasets due to differences in the configuration of quasi-identifiers.

**Table 6:** The features of the de-identified datasets

Dataset numbers	Re-identifi cation risk -before	Re-identi fication risk -after	Re-identi fication risk reduction rate	ARX utility score	EMD	# of records retained for logistic regression	# of predictors retained for logistic regression	Dataset retention ratio
1	0.993	0.064	0.936	0.722	62.346	547	11	0.401
2	0.993	0.076	0.924	0.807	62.559	396	11	0.290
3	0.993	0.064	0.936	0.722	62.346	547	11	0.401
4	0.993	0.076	0.924	0.807	62.559	396	11	0.290
5	0.908	0.044	0.952	0.485	61.746	954	12	0.762
6	0.908	0.059	0.935	0.599	62.017	765	12	0.611
7	0.908	0.000	1.000	1.000	61.118	1119	7	0.522
8	0.908	0.000	1.000	1.000	61.118	1119	7	0.522
9	0.963	0.059	0.939	0.500	61.623	910	12	0.727
10	0.963	0.085	0.911	0.600	61.945	756	12	0.604
11	0.963	0.002	0.998	0.890	62.542	1155	9	0.692
12	0.963	0.002	0.998	0.846	62.737	1155	9	0.692
13	0.135	0.014	0.897	0.449	61.414	1113	13	0.964
14	0.135	0.002	0.986	0.654	61.521	1052	12	0.841
15	0.135	0.014	0.897	0.449	61.414	1113	13	0.964
16	0.135	0.014	0.897	0.449	61.414	1113	13	0.964
17	0.965	0.064	0.934	0.749	63.512	547	11	0.401
18	0.991	0.076	0.924	0.749	62.558	396	11	0.290
19	0.943	0.064	0.932	0.639	63.498	547	11	0.401

*Note.* The number of records in the original dataset: 1155, the number of predictors for logistic regression: 13

Overall, all 19 de-identification scenarios significantly reduced re-identification risk. However, the data transformation processes involved in de-identification led to record suppression and complete masking of variables used as predictors, thereby compromising dataset utility. Notably, except for three datasets (13, 15, 16), which used only *sex* and *age* as quasi-identifiers, there was a loss of one or more predictor variables. Datasets 13, 15, and 16 demonstrated the highest retention ratios and the lowest ARX utility and EMD scores, indicating minimal information loss and the highest similarity to the original dataset, thus reflecting superior dataset utility. They also exhibited the lowest baseline and post-transformation re-identification risks.

Datasets 7 and 8 underwent a transformation under the most complex de-identification scenarios, employing three quasi-identifiers and applying both L-diversity and T-closeness to two sensitive variables. Although these datasets achieved complete re-identification risk reduction, the extensive data transformation allowed only seven predictor variables to be generated. The de-identification scenarios 1 and 3, 2 and 4, and 13, 15, and 16 shared identical configurations of quasi-identifiers but varied in the L-diversity and T-closeness conditions applied to sensitive information, resulting in identical de-identified datasets (see Table 3).

Table 7 details the differences in variable distribution between each transformed dataset and the original dataset. As expected, variables designated as quasi-identifiers underwent the most transformation, leading to significant changes. Variables derived from these quasi-identifiers, such as *sending hospital type, circulatory disease*, and *high priority disease*, also exhibited notable distributional changes.

**Table 7:** The difference in the variable distributions between the de-identified datasets and the original dataset

Dataset numbers														
	Male	Age < 60	Acuity level	Number of consults	Inter hospital communication	Sending hospital location	High priority disease	Neo plastic disease	Circulatory disease	Respi ratory disease	Sending hospital type	Injury-related visits	Treatment outcome: admission	LOS > 6 (outcome variable)
1	***	***			**	***	NA		**		NA	*		
2		***			**	***	NA		***		NA	**		
3	***	***			**	***	NA		**		NA	*		
4		***			**	***	NA		***		NA	**		
5	***	**					NA							
6		**					NA		*					
7	NA	NA					NA	NA	NA	NA				
8	NA	NA					NA	NA	NA	NA				
9	*	***				***					NA			
10		**				***					NA			
11	NA	NA				NA					NA			
12	NA	NA				NA					NA			
13	***													
14	NA													
15	***													
16	***													
17	***				**	***	NA		**		NA	*		
18		***			**	***	NA		***		NA	**		
19					**	***	NA		**		NA	*		

*p < 0.05, **p < 0.01, ***p < 0.001, NA: not comparable because the variable was lost in the de-identified dataset.

### The prediction results

**Fig. 2:**
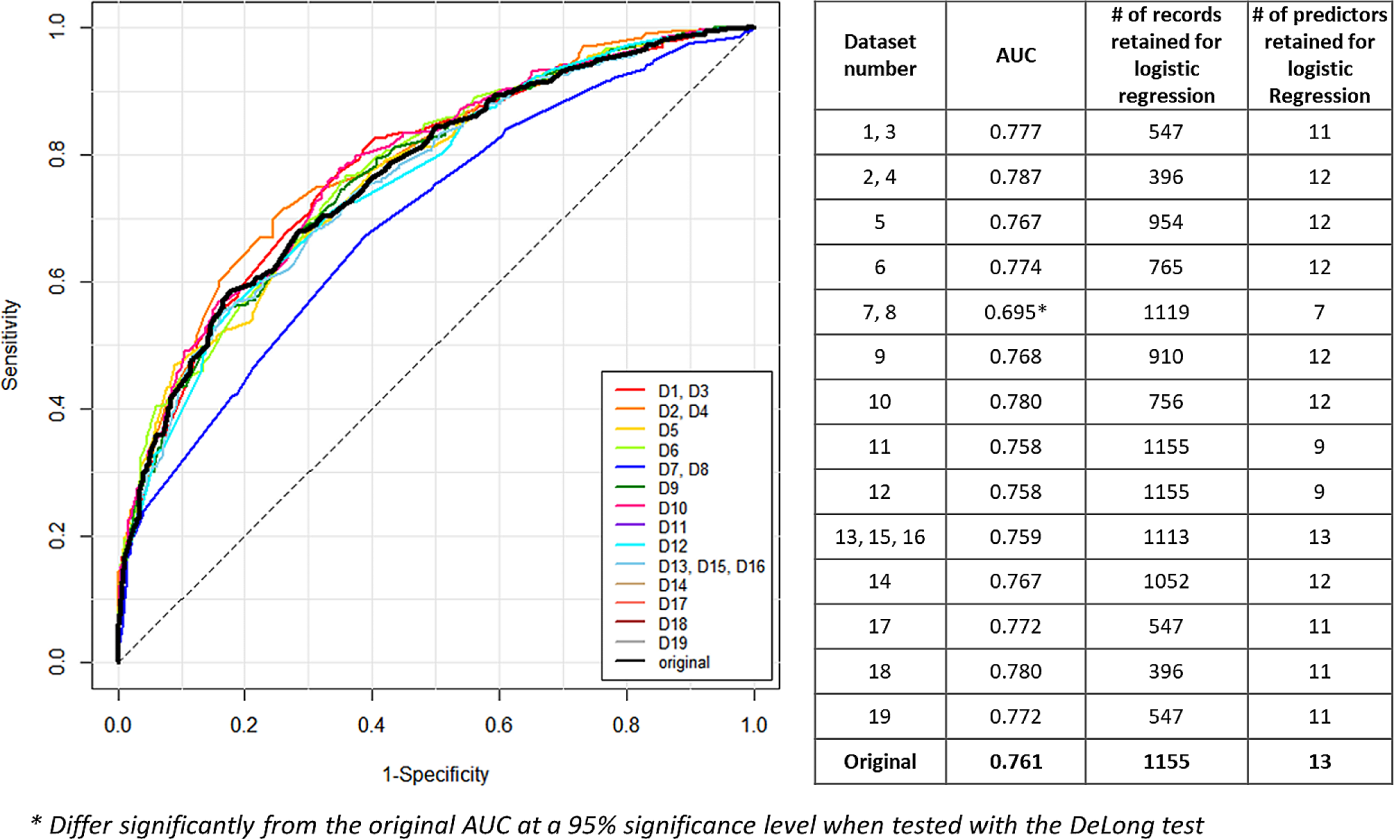
The number of records and predictors included in each model and the model performance

Logistic regression models were developed using both the original dataset and 19 de-identified datasets. The complete masking of variables classified as quasi-identifiers in some de-identified datasets resulted in differences in the number and types of predictors available for constructing the logistic regression models. Additionally, the number of records included in the regression analysis varied due to record suppression associated with the de-identification process. Figure 2 illustrates the ROC curves and the AUC values for all 20 datasets. The AUC values ranged from 0.695 to 0.787. The models generated from datasets 7 and 8, which only retained seven predictors due to extensive data masking, exhibited a statistically significant difference in AUC when compared to the original dataset, with a p-value of 0.002. For the models derived from the other datasets, no significant differences in AUC values were observed.

**Fig. 3:**
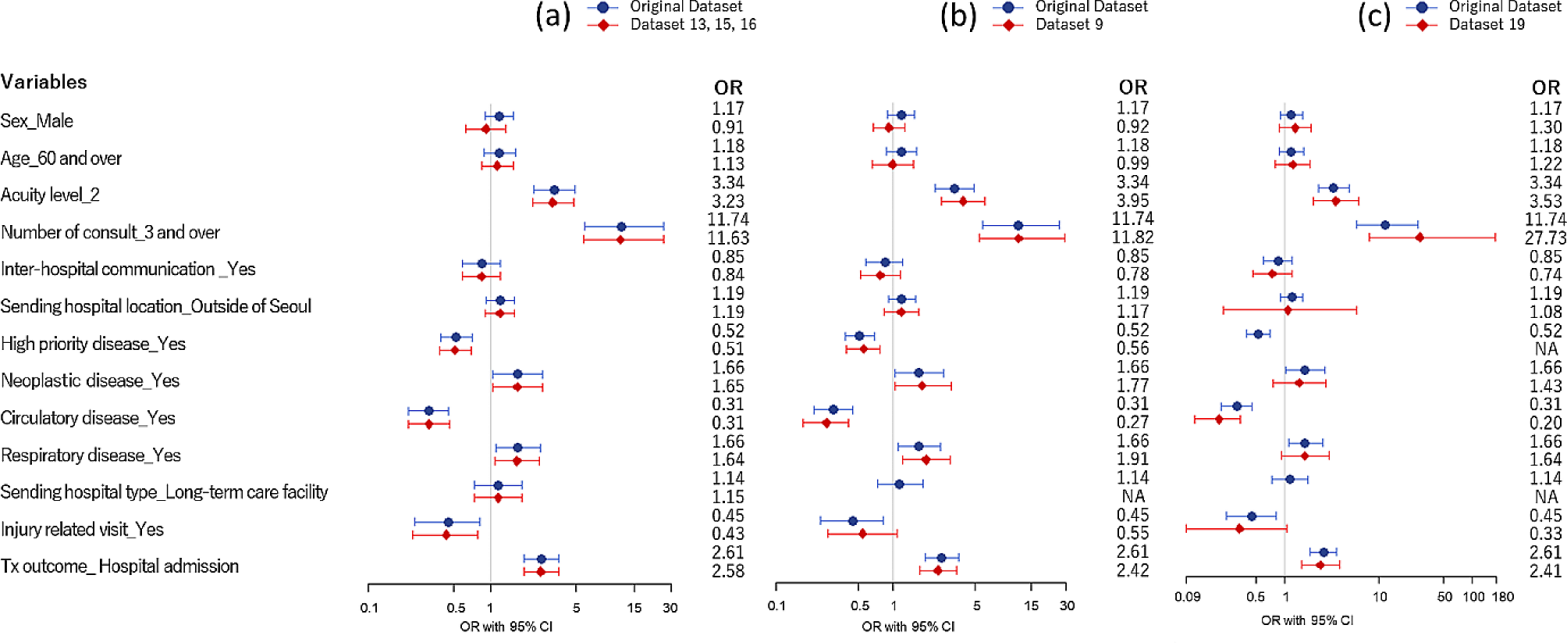
The Odds-Ratios of the predictors from the original dataset and the selected de-identified datasets

Figure 3 displays the Odds Ratios (OR) for predictors from selected datasets. Datasets 13, 15, and 16 were chosen because they retained all 13 predictor variables (Fig. 3(a)). Dataset 9 was selected for having the next highest number of predictors (*N* = 12) and for utilizing three quasi-identifiers: the *sending hospital*, which is identified as the most revealing variable in Table 2, along with *sex* and *age*, which are commonly used as quasi-identifiers (Fig. 3(b)). Dataset 19 was also included because it was configured using only the *sending hospital* and *primary diagnosis* as quasi-identifiers (Fig. 3(c)). The ORs for all 19 datasets are detailed in Additional file 1: Figure S1.

As depicted in Fig. 3(a), the original dataset and de-identified datasets 13, 15, and 16 showed comparable prediction outcomes, with *sex* being the only predictor that displayed an OR notably different from the original dataset; however, it was not statistically significant in either model. Figure 3(b) indicates that the ORs of the 12 predictors in dataset 9 were similar to those in the original dataset, although the OR for injury-related visits became insignificant. In contrast, dataset 19, which excluded two predictors, showed more pronounced differences in the ORs of the 11 remaining predictors (Fig. 3(c)). Additionally, *neoplastic disease* and *respiratory disease*, significant predictors in the original dataset, became insignificant in dataset 9, while *injury-related visits*, previously insignificant, became significant (Fig. 3(c)).

### Data utility vs. data privacy

**Fig. 4:**
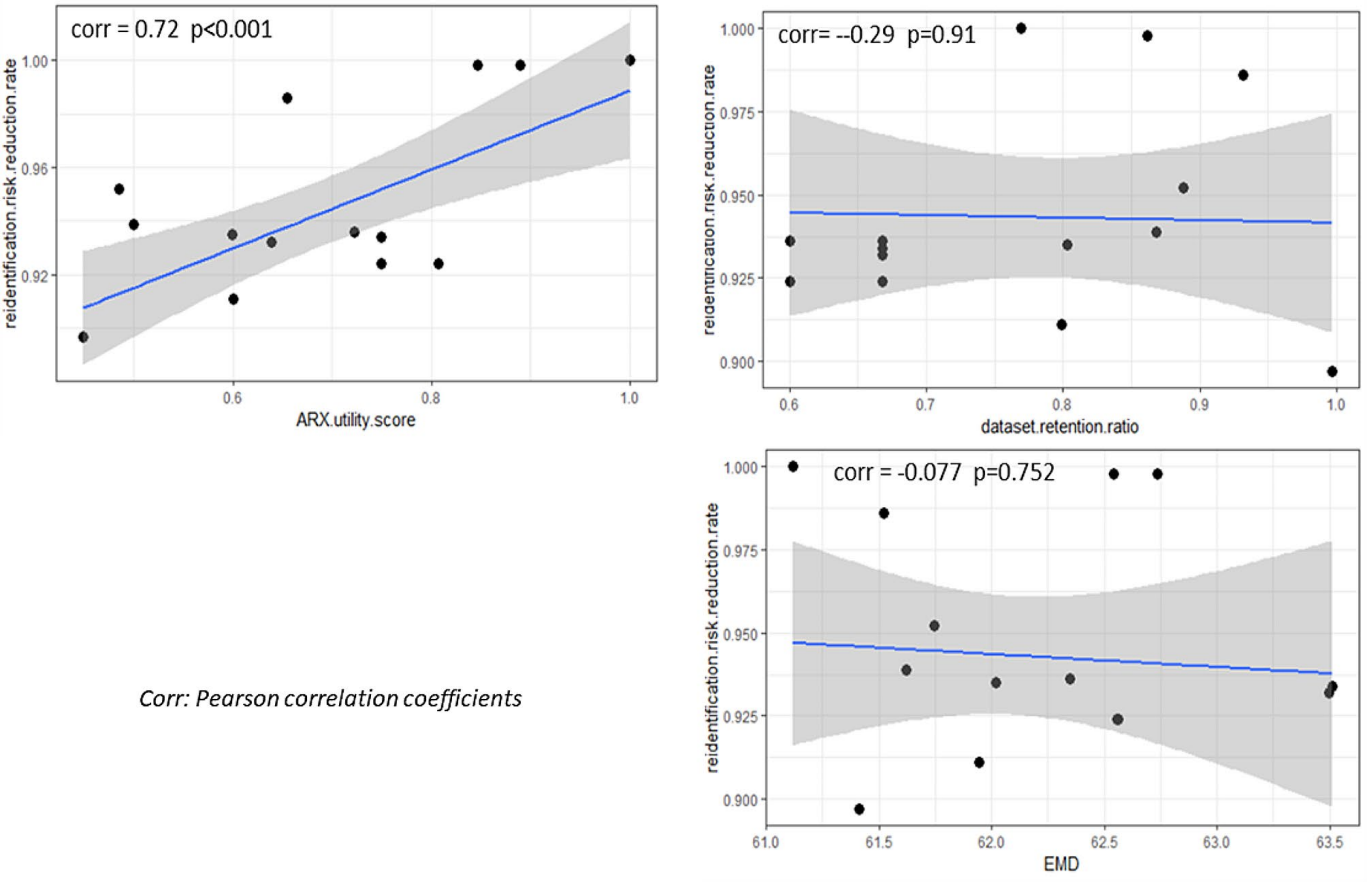
The correlations between re-identification risk reduction and features of the de-identified datasets

Figure 4 presents the correlations between re-identification risk reduction rates, ARX utility scores, EMD, and dataset retention ratios. There is a significant correlation between the re-identification reduction rate and the ARX utility score, indicating that greater reductions in re-identification risk are typically accompanied by larger losses of information. Conversely, the re-identification reduction rate exhibits a slight negative correlation with both EMD and dataset retention ratio; however, these correlations are not statistically significant.

## Discussion

This study tested various de-identification strategies on a clinical dataset, adjusting the number and types of quasi-identifiers and sensitive information, and configuring K-anonymity, L-diversity, and T-closeness in diverse ways. It aimed to address gaps left by earlier studies that utilized simplistic data use cases and de-identification configurations [28, 34, 35].

The results indicated that de-identification led to the suppression of records and variables, precluding the replication of analyses performed on the original dataset. Consequently, logistic regression models for predicting ED LOS yielded differing conclusions based on the de-identification approach, as illustrated in Fig. 3. This highlights the need for the evolution of privacy technologies that maintain data integrity. Additionally, it cautions data users about potential biases introduced when working with de-identified datasets.

The study found optimal data utility when only *sex* and *age* were classified as quasi-identifiers, maintaining all variables and losing only six records. This configuration also significantly reduced the baseline re-identification risk, albeit *sex* and *age* by themselves did not strongly individualize records. However, this configuration did not account for the additional re-identification risk posed by the *sending hospital* and *primary diagnosis*, both of which were considered the most identifying variables in the dataset (Table 2). To eliminate any alterations to *sex* and *age*—key variables for clinical research—we examined the impact of designating only the *sending hospital* and *primary diagnosis* as quasi-identifiers (dataset 19). This strategy greatly reduced the chance of re-identification but at a considerable cost to data utility, resulting in the loss of over half the dataset and two predictor variables: the *sending hospital type* and *high priority disease*.

Seeking a compromise, datasets 5–12 incorporated *sex*, *age*, and either *sending hospital* or *primary diagnosis* as quasi-identifiers. In this series, datasets 7 and 8 achieved zero re-identification risk post-de-identification but sacrificed nearly half of the predictor variables. Datasets 11 and 12, while managing to retain all records, were considered less favorable due to the loss of four predictor variables. Datasets 5 and 6 struck a more acceptable balance, offering substantial re-identification risk reduction, retaining over 78% of records, and sacrificing only one predictor variable. Although dataset 5 had marginally better scores for risk reduction and data utility, dataset 6 was preferred because it retained information on *high priority disease*, a key predictor of ED LOS.

In this study, three different data utility metrics were examined, but only the ARX utility score exhibited a statistically significant correlation with the re-identification risk reduction rate. The EMD and dataset retention ratio both showed minor negative correlations with re-identification risk reduction; however, these were not statistically significant. This could suggest that the structural aspects of a dataset may not alone be adequate for assessing its utility, although further studies with a broader array of datasets would be required to substantiate this preliminary indication.

The scope of this research was limited to a single use case, analyzing data obtained from one hospital. Moreover, the range of de-identification scenarios tested did not encompass the full spectrum of complex configurations that could be employed. Despite these constraints, the research offers valuable insights into the nuanced interplay between data de-identification processes and data utility. It contributes to the ongoing conversation about how to approach data privacy in a way that still enables effective data usage.

## Conclusion

As health data analysis grows more critical, so does the imperative to devise effective methods for ensuring data privacy. While established guidelines [47] offer a foundation for the de-identification of datasets, crafting a dataset that maintains a high level of privacy without unduly compromising its utility remains a nuanced challenge. It demands a thorough grasp of the data’s intended application. Incorporating input from data users during the de-identification process and considering the variety of potential data use cases could prove beneficial in finding a workable tradeoff between data privacy and utility.

### Electronic supplementary material

Below is the link to the electronic supplementary material.

Supplementary Material 1

## Data Availability

The clinical dataset used in this study is not made available due to the sensitive nature of clinical data. However, de-identified analytic datasets are available upon reasonable request from the corresponding author and with permission of Seoul National University Hospital.

## Abbreviations

AUC: Area Under the receiver operating characteristic (ROC) Curve
DP: Differential Privacy
ED: Emergency Department
EMD: Earth Mover’s Distance
HE: Homomorphic Encryption
ICD: International Classification of Disease
LOS: Length Of Stay
ROC: Receiver Operating Characteristic
