# Supplementary material for: Exploring the tradeoff between data privacy and utility with a clinical data analysis use case
Source: BMC Med Inform Decis Mak. 2024 May 30;24:147. doi: 10.1186/s12911-024-02545-9 (PMC11137882; doi:10.1186/s12911-024-02545-9)

Additional file 1

**Figure S1. The ORs of the predictors from all 19 de-identified datasets and original dataset**


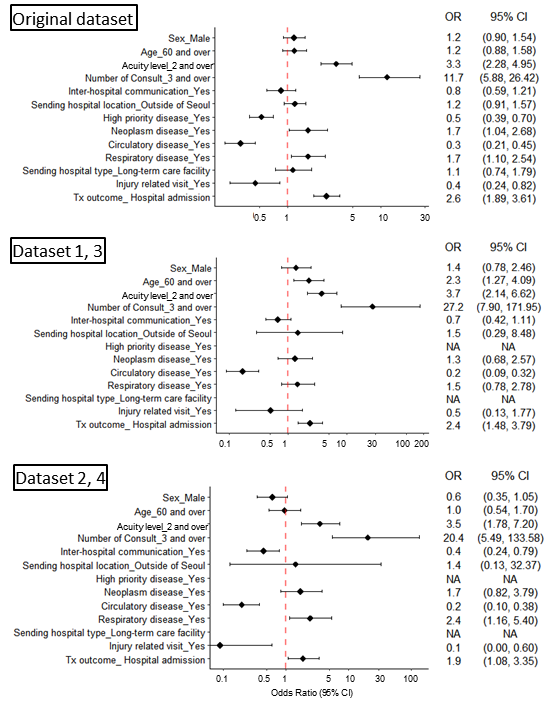


**Figure S1. (Continued)The ORs of the predictors from all 19 de-identified datasets and original dataset**


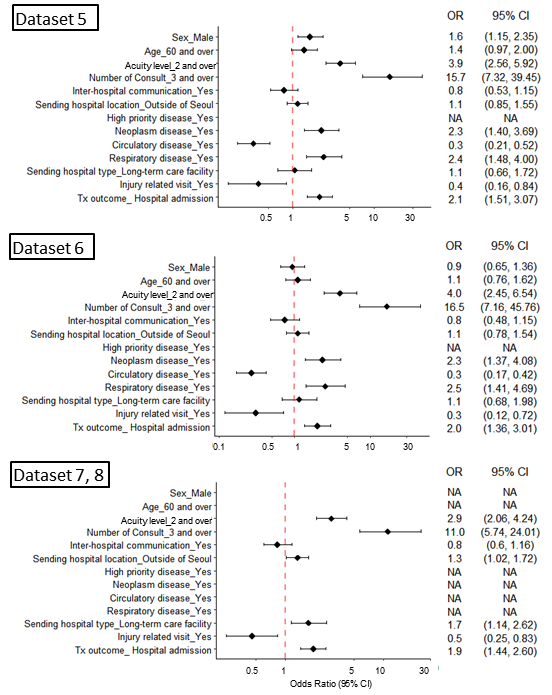


**Figure S1. (Continued)The ORs of the predictors from all 19 de-identified datasets and original dataset**


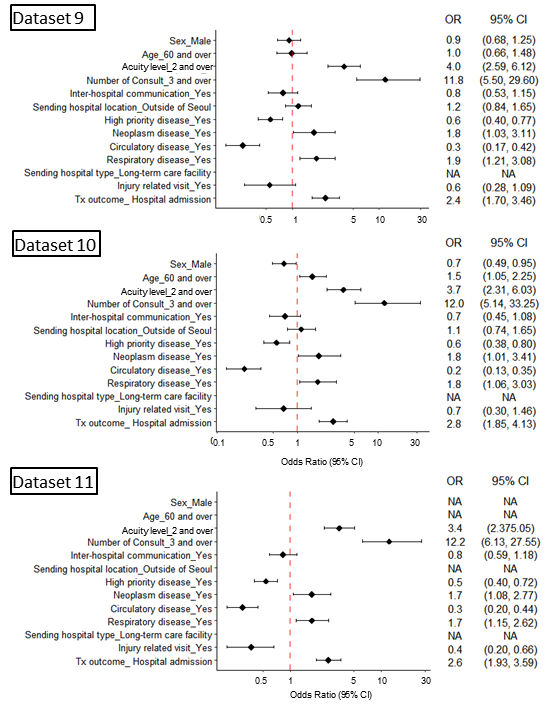


**Figure S1. (Continued)The ORs of the predictors from all 19 de-identified datasets and original dataset**


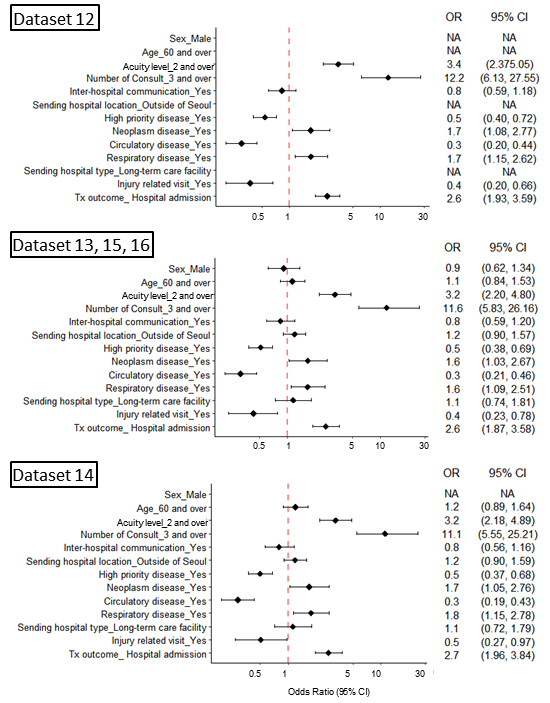


**Figure S1. (Continued)The ORs of the predictors from all 19 de-identified datasets and original dataset**


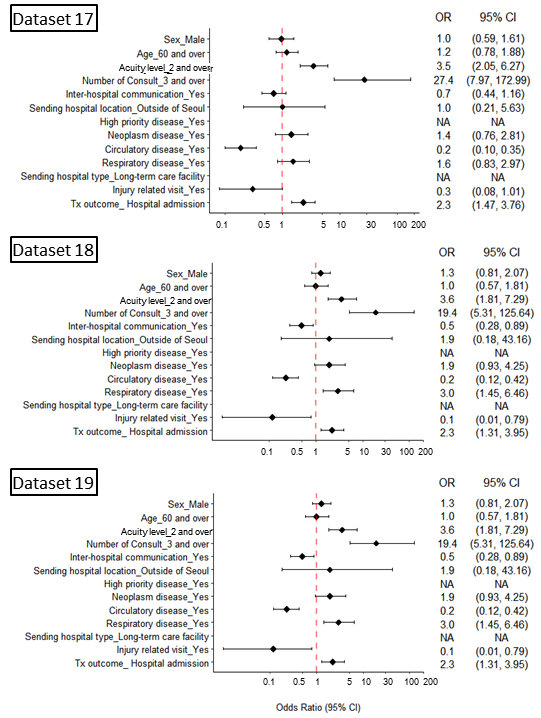

Supplement: Supplementary file 1 — Supplementary Material 1 [file 12911_2024_2545_MOESM1_ESM.docx]
